# Supplementary material for: Current and Expected Trends for the Marine Chitin/Chitosan and Collagen Value Chains
Source: Mar Drugs. 2023 Nov 23;21(12):605. doi: 10.3390/md21120605 (PMC10744996; doi:10.3390/md21120605)
Supplement: Supplementary file 1 [file marinedrugs-21-00605-s001.zip › Table S1.pdf]

Table S1 - Keywords Combinations submitted to Scopus and WoS and results without duplicates

Keyword Combinations	Number of papers
Chitin AND Chitosan AND (Aquaculture OR Fisheries OR Industr*) AND (Crustaceans OR Shellfish OR Marine)	453
Chitin AND Chitosan AND (Value Chain OR Market OR Market Demand OR ROI) AND (Crustaceans OR Shellfish OR Marine)	38
Chitin AND Chitosan AND Bio* Waste AND (Marine Resources OR Crustaceans OR Shellfish OR Marine)	38
(Collagen hydrolysate OR Collagen) AND (Aquaculture OR Fisheries OR Industr*) AND (Crustaceans OR Shellfish OR Marine)	334
(Collagen hydrolysate OR Collagen) AND (Value Chain OR Market OR Market Demand OR ROI) AND (Crustaceans OR Shellfish OR Marine)	42
(Collagen hydrolysate OR Collagen) AND Bio* Waste AND (Crustaceans OR Shellfish OR Marine)	12
	917
