# Supplementary material for: Current and Expected Trends for the Marine Chitin/Chitosan and Collagen Value Chains
Source: Mar Drugs. 2023 Nov 23;21(12):605. doi: 10.3390/md21120605 (PMC10744996; doi:10.3390/md21120605)
Supplement: Supplementary file 1 [file marinedrugs-21-00605-s001.zip › Table S2.pdf]

Table S2 - Assessed publications

Abdelmalek, B. E., Sila, A., Haddar, A., Bougatef, A., & Ayadi, M. A. (2017). β-Chitin and chitosan from squid gladius: Biological activities of chitosan and its application as clarifying agent for apple juice. International Journal of Biological Macromolecules, 104, 953–962. https://doi.org/10.1016/j.ijbiomac.2017.06.107
Abdullah, T., Gzara, L., Simonetti, G., Alshahrie, A., Salah, N., Morganti, P., Chianese, A., Fallahi, A., Tamayol, A., Bencherif, S., & Memic, A. (2018). The Effect of Poly (Glycerol Sebacate) Incorporation within Hybrid Chitin–Lignin Sol–Gel Nanofibrous Scaffolds. Materials, 11(3), 451. https://doi.org/10.3390/ma11030451
Aboudamia, F. Z., Kharroubi, M., Neffa, M., Aatab, F., Hanoune, S., Bouchdoug, M., & Jaouad, A. (2020). Potential of discarded sardine scales (Sardina pilchardus) as chitosan sources. Journal of the Air and Waste Management Association, 70(11), 1186–1197. https://doi.org/10.1080/10962247.2020.1813840
Acharya, P. P., Kupendra, M. H., Fasim, A., More, S. S., & Murthy, V. K. (2022). A comparative assessment of collagen type 1 from silver carp (fresh water) and milk shark(marine) fish waste. 3 Biotech, 12(3). https://doi.org/10.1007/s13205-022-03114-5
Addad, S., Exposito, J.-Y., Faye, C., Ricard-Blum, S., & Lethias, C. (2011). Isolation, characterization and biological evaluation of jellyfish collagen for use in biomedical applications. Marine Drugs, 9(6), 967–983. https://doi.org/10.3390/md9060967
Águila-Almanza, E., Low, S. S. S., Hernández-Cocoletzi, H., Atonal-Sandoval, A., Rubio-Rosas, E., Violante-González, J., & Show, P. L. L. (2021). Facile and green approach in managing sand crab carapace biowaste for obtention of high deacetylation percentage chitosan. Journal of Environmental Chemical Engineering, 9(3). https://doi.org/10.1016/j.jece.2021.105229
Ahmed, A., Hassan, A., & Nour, M. (2021). Utilization of chitosan extracted from shrimp shell waste in wastewater treatment as low cost biosorbent. Egyptian Journal of Chemistry, 0–0. https://doi.org/10.21608/ejchem.2020.43166.2871
Ahmed, M., Anand, A., Verma, A. K., & Patel, R. (2022). In-vitro self-assembly and antioxidant properties of collagen type I from Lutjanus erythropterus, and Pampus argenteus skin. Biocatalysis and Agricultural Biotechnology, 43. https://doi.org/10.1016/j.bcab.2022.102412
Akita, M., Kono, T., Lloyd, K., Mitsui, T., Morioka, K., & Adachi, K. (2019). Biochemical study of type I collagen purified from skin of warm sea teleost Mahi mahi (Coryphaena hippurus), with a focus on thermal and physical stability. Journal of Food Biochemistry, 43(11). https://doi.org/10.1111/jfbc.13013
Al Sagheer, F. A., Al-Sughayer, M. A., Muslim, S., & Elsabee, M. Z. (2009). Extraction and characterization of chitin and chitosan from marine sources in Arabian Gulf. Carbohydrate Polymers, 77(2), 410–419. https://doi.org/10.1016/J.CARBPOL.2009.01.032
Al-Ali, R. M., Al-Hilifi, S. A., & Rashed, M. M. A. (2021). Fabrication, characterization, and anti-free radical performance of edible packaging-chitosan film synthesized from shrimp shell incorporated with ginger essential oil.

Journal of Food Measurement and Characterization, 15(4), 2951–2962. https://doi.org/10.1007/s11694-021-00875-0
Aldebs, A. I., Abdulameer, H. A., & Abudken, A. M. (2022). Isolation and Characterization of Collagen Extracted from Fish Scales and Applied as Anti-TNFα Protein. Bahrain Medical Bulletin, 44(4), 1177–1180. https://www.scopus.com/inward/record.uri?eid=2-s2.0-85146392330&partnerID=40&md5=78304c47ac7838d1bd0a1159622c9bdd
Alves, A. L., Marques, A. L. P., Martins, E., Silva, T. H., & Reis, R. L. (2017). Cosmetic potential of Marine fish skin collagen. Cosmetics, 4(4). https://doi.org/10.3390/cosmetics4040039
Alves, H. J., Furman, M., Kugelmeier, C. L., De Oliveira, C. R., Bach, V. R., Lupatini, K. N., Neves, A. C., & Arantes, M. K. (2017). Effect of shrimp shells milling on the molar mass of chitosan. Polimeros, 27(1), 41–47. https://doi.org/10.1590/0104-1428.2354
Amer, M. S., & Ibrahim, H. A. H. (2019). Chitosan from marine-derived Penicillum spinulosum MH2 cell wall with special emphasis on its antimicrobial and antifouling properties. The Egyptian Journal of Aquatic Research, 45(4), 359–365. https://doi.org/10.1016/J.EJAR.2019.11.007
Amer, O. A., Ali, S. S., Azab, M., El-Shouny, W. A., Sun, J., & Mahmoud, Y. A.-G. (2022). Exploring new marine bacterial species, Alcaligenes faecalis Alca F2018 valued for bioconversion of shrimp chitin to chitosan for concomitant biotechnological applications. International Journal of Biological Macromolecules, 196, 35–45. https://doi.org/10.1016/j.ijbiomac.2021.12.033
Amiri, H., Aghbashlo, M., Sharma, M., Gaffey, J., Manning, L., Basri, S. M. M., Kennedy, J. F., Gupta, V. K., & Tabatabaei, M. (2022). Chitin and chitosan derived from crustacean waste valorization streams can support food systems and the UN Sustainable Development Goals. NATURE FOOD, 3(10), 822–828. https://doi.org/10.1038/s43016-022-00591-y
Aneesh, P. A., Anandan, R., Kumar, L. R. G., Ajeeshkumar, K. K., Kumar, K. A., & Mathew, S. (2020). A step to shell biorefinery—Extraction of astaxanthin-rich oil, protein, chitin, and chitosan from shrimp processing waste. Biomass Conversion and Biorefinery. https://doi.org/10.1007/s13399-020-01074-5
Anouar, A., Grirrane, A., Álvarez, E., Katir, N., Primo, A., Garcia, H., & El Kadib, A. (2022). Nanosized copper stabilized on ternary P, N, S-doped graphene from chitosan shellfish waste: preparation and catalysis of single and double A3-type amine coupling. Materials Today Sustainability, 18. https://doi.org/10.1016/j.mtsust.2022.100109
Apetroaei, M., Manea, A.-M., Tihan, G., Zgârian, R., Schroder, V., & Rău, I. (2017). Improved method of chitosan extraction from different crustacean species of Romanian black sea coast. UPB Scientific Bulletin, Series B: Chemistry and Materials Science, 79(1), 25–36.
Arias-Moscoso, J. L., Soto-Valdez, H., Plascencia-Jatomea, M., Vidal-Quintanar, R.-L., Rouzaud-Sández, O., & Ezquerra-Brauer, J. M. (2011). Composites of chitosan with acid-soluble collagen from jumbo squid (Dosidicus gigas) by-products. Polymer International, 60(6), 924–931. https://doi.org/10.1002/pi.3048

Arulmoorthy, M. P., Anbarasi, G., Srinivasan, M., & Vishnupriya, B. (2022). Biosynthesis and characterization of chitosan based hydrogel: A potential in vitro wound healing agent. In MATERIALS TODAY-PROCEEDINGS (Vol. 48, Issue International Conference on Impact of Innovations in Science and Technology for Societal Development (IISTSD)-Materials Science, pp. 263–275). https://doi.org/10.1016/j.matpr.2020.07.186 WE - Conference Proceedings Citation Index - Science (CPCI-S)
Babeanu, N., Radu, N., Enascuta, C. E., Alexandrescu, E., Ganciarov, M., Mohammed, M. S. O., Suica-Bunghez, I. R., Senin, R., Ursu, M., & Bostan, M. (2022). Obtaining and Characterizing Composite Biomaterials of Animal Resources with Potential Applications in Regenerative Medicine. POLYMERS, 14(17). https://doi.org/10.3390/polym14173544 WE - Science Citation Index Expanded (SCI-EXPANDED)
Balde, A., Hasan, A., Joshi, I., & Nazeer, R. A. (2020). Preparation and optimization of chitosan nanoparticles from discarded squilla (Carinosquilla multicarinata) shells for the delivery of anti-inflammatory drug: Diclofenac. Journal of the Air and Waste Management Association, 70(12), 1227–1235. https://doi.org/10.1080/10962247.2020.1727588
Balitaan, J. N. I., Yeh, J. M., & Santiago, K. S. (2020). Marine waste to a functional biomaterial: Green facile synthesis of modified-β-chitin from Uroteuthis duvauceli pens (gladius). International Journal of Biological Macromolecules, 154, 1565–1575. https://doi.org/10.1016/J.IJBIOMAC.2019.11.041
Banerjee, P., & Das, J. (2017). Biomimetic synthesis of nanocrystalline hydroxyapatite from sharkskin collagen. Bioinspired, Biomimetic and Nanobiomaterials, 7(1), 27–36. https://doi.org/10.1680/jbibn.16.00018
Barber, P. S., Griggs, C. S., Bonner, J. R., & Rogers, R. D. (2013). Electrospinning of chitin nanofibers directly from an ionic liquid extract of shrimp shells. Green Chemistry, 15(3), 601. https://doi.org/10.1039/c2gc36582k
Bardakova, K. N., Akopova, T. A., Kurkov, A. V., Goncharuk, G. P., Butnaru, D. V., Burdukovskii, V. F., Antoshin, A. A., Farion, I. A., Zharikova, T. M., Shekhter, A. B., Yusupov, V. I., Timashev, P. S., & Rochev, Y. A. (2019). From Aggregates to Porous Three-Dimensional Scaffolds through a Mechanochemical Approach to Design Photosensitive Chitosan Derivatives. Marine Drugs, 17(1), 48. https://doi.org/10.3390/md17010048
Batista, M. P., Fernández, N., Gaspar, F. B., Bronze, M. D. R., & Duarte, A. R. C. (2022). Extraction of Biocompatible Collagen From Blue Shark Skins Through the Conventional Extraction Process Intensification Using Natural Deep Eutectic Solvents. Frontiers in Chemistry, 10. https://doi.org/10.3389/fchem.2022.937036
Bazargan-Lari, R., Bahrololoom, M. E., & Nemati, A. (2011). Sorption behavior of Zn (II) ions by low cost and biological natural hydroxyapatite/chitosan composite from industrial waste water. Journal of Food, Agriculture and Environment, 9(3–4), 892–897.
Beaney, P., Lizardi-Mendoza, J., & Healy, M. (2005). Comparison of chitins produced by chemical and bioprocessing methods. Journal of Chemical Technology and Biotechnology, 80(2), 145–150. https://doi.org/10.1002/jctb.1164
Bedekar, A. N., Pise, A. C., Thatte, C. S., & Rathnam, M. V. (2010). Study on optimization of carboxymethylation of chitosan obtained from squilla chitin. Asian Journal of Chemistry, 22(10), 7675–7682.

Bernardi, F., Zadinelo, I. V., Alves, H. J., Meurer, F., & dos Santos, L. D. (2018). Chitins and chitosans for the removal of total ammonia of aquaculture effluents. Aquaculture, 483, 203–212. https://doi.org/10.1016/j.aquaculture.2017.10.027
Bisht, M., Martins, M., Dias, A. C. R. V., Ventura, S. P. M., & Coutinho, J. A. P. (2021). Uncovering the potential of aqueous solutions of deep eutectic solvents on the extraction and purification of collagen type I from Atlantic codfish (Gadus morhua). Green Chemistry, 23(22), 8940–8948. https://doi.org/10.1039/d1gc01432c
Blanco, M., Vázquez, J. A., Pérez-Martín, R. I., & Sotelo, C. G. (2019). Collagen extraction optimization from the skin of the small-spotted catshark (S. Canicula) by response surface methodology. Marine Drugs, 17(1). https://doi.org/10.3390/md17010040
Borzacchiello, A., Ambrosio, L., Netti, P. A., Nicolais, L., Peniche, C., Gallardo, A., & San Roman, J. (2001). Chitosan-based hydrogels: Synthesis and characterization. Journal of Materials Science: Materials in Medicine, 12(10–12), 861–864. https://doi.org/10.1023/A:1012851402759
Bradić, B., Novak, U., & Likozar, B. (2019). Crustacean shell bio-refining to chitin by natural deep eutectic solvents. Green Processing and Synthesis, 9(1), 13–25. https://doi.org/10.1515/gps-2020-0002
Buscaglia, M., Guérard, F., Roquefort, P., Aubry, T., Fauchon, M., Toueix, Y., Stiger-Pouvreau, V., Hellio, C., & Le Blay, G. (2022). Mechanically Enhanced Salmo salar Gelatin by Enzymatic Cross-linking: Premise of a Bioinspired Material for Food Packaging, Cosmetics, and Biomedical Applications. Marine Biotechnology, 24(4), 801–819. https://doi.org/10.1007/s10126-022-10150-y
Cahú, T. B., Santos, S. D., Mendes, A., Córdula, C. R., Chavante, S. F., Carvalho, L. B., Nader, H. B., & Bezerra, R. S. (2012). Recovery of protein, chitin, carotenoids and glycosaminoglycans from Pacific white shrimp (Litopenaeus vannamei) processing waste. Process Biochemistry, 47(4), 570–577. https://doi.org/10.1016/j.procbio.2011.12.012
Cardozo, F. A., Facchinatto, W. M., Colnago, L. A., Campana-Filho, S. P., & Pessoa, A. (2019). Bioproduction of N-acetyl-glucosamine from colloidal α-chitin using an enzyme cocktail produced by Aeromonas caviae CHZ306. World Journal of Microbiology and Biotechnology, 35(8), 114. https://doi.org/10.1007/s11274-019-2694-x
Carrera, M., Ezquerra-Brauer, J. M., & Aubourg, S. P. (2020). Characterization of the jumbo squid (dosidicus gigas) skin by-product by shotgun proteomics and protein-based bioinformatics. Marine Drugs, 18(1). https://doi.org/10.3390/md18010031
Carvalho, A. M., Marques, A. P., Silva, T. H., & Reis, R. L. (2018). Evaluation of the potential of collagen from codfish skin as a biomaterial for biomedical applications. Marine Drugs, 16(12). https://doi.org/10.3390/md16120495
Casanova, F., Mohammadifar, M. A., Jahromi, M., Petersen, H. O., Sloth, J. J., Eybye, K. L., Kobbelgaard, S., Jakobsen, G., & Jessen, F. (2020). Physico-chemical, structural and techno-functional properties of gelatin from saithe (Pollachius virens) skin. International Journal of Biological Macromolecules, 156, 918–927. https://doi.org/10.1016/J.IJBIOMAC.2020.04.047

Chen, B., Yu, L., Wu, J., Qiao, K., Cui, L., Qu, H., Su, Y., Cai, S., Liu, Z., & Wang, Q. (2022). Effects of Collagen Hydrolysate From Large Hybrid Sturgeon on Mitigating Ultraviolet B-Induced Photodamage. Frontiers in Bioengineering and Biotechnology, 10. https://doi.org/10.3389/fbioe.2022.908033

Chen, S., Yang, Q., Chen, X., Tian, Y., Liu, Z., & Wang, S. (2020). Bioactive peptides derived from crimson snapper and: In vivo anti-aging effects on fat diet-induced high fat Drosophila melanogaster. Food and Function, 11(1), 524–533. https://doi.org/10.1039/c9fo01414d

Chiarelli, P. G., Pegg, R. B., Dev Kumar, G., & Mis Solval, K. (2021). Exploring the feasibility of developing novel gelatin powders from salted, dried cannonball jellyfish (Stomolophus meleagris). Food Bioscience, 44. https://doi.org/10.1016/j.fbio.2021.101397

Chik, C., Kamaruzzan, A. S., Rahim, A. I. A., Lananan, F., Endut, A., Aslamyah, S., & Kasan, N. A. (2023). Extraction and Characterization of Litopenaeus vannamei's Shell as Potential Sources of Chitosan Biopolymers. JOURNAL OF RENEWABLE MATERIALS, 11(3), 1181–1197. https://doi.org/10.32604/jrm.2023.022755

Cho, J.-K., Jin, Y.-G., Rha, S.-J., Kim, S.-J., & Hwang, J.-H. (2014). Biochemical characteristics of four marine fish skins in Korea. Food Chemistry, 159, 200–207. https://doi.org/10.1016/j.foodchem.2014.03.012

Chung, Y.-C. (2006). Improvement of aquaculture wastewater using chitosan of different degrees of deacetylation. Environmental Technology, 27(11), 1199–1208. https://doi.org/10.1080/09593332708618734

Cogollo-Herrera, K., Bonfante-Álvarez, H., De Ávila-Montiel, G., Barros, A. H., & González-Delgado, Á. D. (2018). Techno-economic sensitivity analysis of large scale chitosan production process from shrimp shell wastes. Chemical Engineering Transactions, 70, 2179–2184. https://doi.org/10.3303/CET1870364

Coughlin, R. W., Deshaies, M. R., & Davis, E. M. (1990). Chitosan in crab shell wastes purifies electroplating wastewater. Environmental Progress, 9(1), 35–39. https://doi.org/10.1002/ep.670090116

Cutajar, N., Lia, F., Deidun, A., Galdies, J., Arizza, V., & Zammit Mangion, M. (2022). Turning Waste into A Resource: Isolation and Characterization of High-Quality Collagen and Oils from Atlantic Bluefin Tuna Discards. Applied Sciences (Switzerland), 12(3). https://doi.org/10.3390/app12031542

Das, J., Dey, P., Chakraborty, T., Saleem, K., Nagendra, R., & Banerjee, P. (2018). Utilization of marine industry waste derived collagen hydrolysate as peroxide inhibition agents in lipid-based food. Journal of Food Processing and Preservation, 42(2). https://doi.org/10.1111/jfpp.13430

Dave, D., Liu, Y., Clark, L., Dave, N., Trenholm, S., & Westcott, J. (2019). Availability of marine collagen from Newfoundland fisheries and aquaculture waste resources. Bioresource Technology Reports, 7. https://doi.org/10.1016/j.biteb.2019.100271

Dawood, M. A. O., Gewaily, M. S., Soliman, A. A., Shukry, M., Amer, A. A., Younis, E. M., Abdel-Warith, A.-W. A., Van Doan, H., Saad, A. H., Aboubakr, M., Abdel-Latif, H. M. R., & Fadl, S. E. (2020). Marine-Derived

Chitosan Nanoparticles Improved the Intestinal Histo-Morphometrical Features in Association with the Health and Immune Response of Grey Mullet (Liza ramada). Marine Drugs, 18(12). https://doi.org/10.3390/md18120611

de la Paz, N., Fernández, M., López, O., Garcia, C., Nogueira, A., Torres, L., Turiño, W., & Heinämäki, J. (2021). Spray drying of chitosan acid salts: Process development, scaling up and physicochemical material characterization. Marine Drugs, 19(6). https://doi.org/10.3390/md19060329

Dehghani, M. H., Dehghan, A., & Najafpoor, A. (2017). Removing Reactive Red 120 and 196 using chitosan/zeolite composite from aqueous solutions: Kinetics, isotherms, and process optimization. Journal of Industrial and Engineering Chemistry, 51, 185–195. https://doi.org/10.1016/j.jiec.2017.03.001

Devi, R., & Dhamodharan, R. (2018). Pretreatment in Hot Glycerol for Facile and Green Separation of Chitin from Prawn Shell Waste. ACS Sustainable Chemistry & Engineering, 6(1), 846–853. https://doi.org/10.1021/acssuschemeng.7b03195

Di Benedetto, C., Barbaglio, A., Martinello, T., Alongi, V., Fassini, D., Cullorà, E., Patruno, M., Bonasoro, F., Barbosa, M. A., Carnevali, M. D. C., Carnevali, M. D. C., & Sugni, M. (2014). Production, characterization and biocompatibility of marine collagen matrices from an alternative and sustainable source: The sea urchin Paracentrotus lividus. Marine Drugs, 12(9), 4912–4933. https://doi.org/10.3390/md12094912

do Vale, D. A., Vieira, C. B., Vidal, M. F., Claudino, R. L., Andrade, F. K., Sousa, J. R., Souza Filho, M. S. M., da Silva, A. L. C., & de Souza, B. W. S. (2021). Chitosan-Based Edible Films Produced from Crab-Uçá (Ucides cordatus) Waste: Physicochemical, Mechanical and Antimicrobial Properties. Journal of Polymers and the Environment, 29(3), 694–706. https://doi.org/10.1007/s10924-020-01913-6

Duasa, J., Husin, A. M., Asmy Mohd Thas Thaker, M., & Rahman, M. P. (2021). An alternative source of collagen for Muslim consumers: halal and environmental concerns. Journal of Islamic Marketing. https://doi.org/10.1108/JIMA-09-2020-0268

El Harmoudi, H., El Gaini, L., Daoudi, E., Rhazi, M., Boughaleb, Y., El Mhammedi, M. A., Migalska-Zalas, A., & Bakasse, M. (2014). Removal of 2,4-D from aqueous solutions by adsorption processes using two biopolymers: Chitin and chitosan and their optical properties. Optical Materials, 36(9), 1471–1477. https://doi.org/10.1016/j.optmat.2014.03.040

El-Beltagi, H. S., El-Mahdy, O. M., Mohamed, H. I., & El-Ansary, A. E. (2022). Antioxidants, Antimicrobial, and Anticancer Activities of Purified Chitinase of Talaromyces funiculosus Strain CBS 129594 Biosynthesized Using Crustacean Bio-Wastes. AGRONOMY-BASEL, 12(11). https://doi.org/10.3390/agronomy12112818 WE - Science Citation Index Expanded (SCI-EXPANDED)

Elwakeel, K. Z., Al-Bogami, A. S., & Elgarahy, A. M. (2018). Efficient Retention of Chromate from Industrial Wastewater onto a Green Magnetic Polymer Based on Shrimp Peels. Journal of Polymers and the Environment, 26(5), 2018–2029. https://doi.org/10.1007/s10924-017-1096-0

Eulálio, H. Y. C., Vieira, M., Fideles, T. B., Tomás, H., Silva, S. M. L., Peniche, C. A., & Fook, M. V. L. (2020). Physicochemical properties and cell viability of shrimp chitosan films as affected by film casting solvents. I-potential use as wound dressing. Materials, 13(21), 1–18. https://doi.org/10.3390/ma13215005

Fabbricino, M., & Gallo, R. (2010). Chromium removal from tannery wastewater using ground shrimp shells. Desalination and Water Treatment, 23(1–3), 194–198. https://doi.org/10.5004/dwt.2010.2020
Fan, R., Zhou, D., & Cao, X. (2020). Evaluation of oat β-glucan-marine collagen peptide mixed gel and its application as the fat replacer in the sausage products. PLoS ONE, 15(5). https://doi.org/10.1371/journal.pone.0233447
Fernández-Marín, R., Hernández-Ramos, F., Salaberria, A. M., Andrés, M. Á., Labidi, J., & Fernandes, S. C. M. (2021). Eco-friendly isolation and characterization of nanochitin from different origins by microwave irradiation: Optimization using response surface methodology. International Journal of Biological Macromolecules, 186, 218–226. https://doi.org/10.1016/j.ijbiomac.2021.07.048
Fontana, R., Marconi, P. C. R., Caputo, A., & Gavalyan, V. B. (2022). Novel Chitosan-Based Schiff Base Compounds: Chemical Characterization and Antimicrobial Activity. Molecules, 27(9). https://doi.org/10.3390/molecules27092740
Gadgey, K. K., & Dey, S. (2017). Development of chitin and chitosan from narmada riverside crab shells. International Journal of Mechanical Engineering and Technology, 8(7), 298–307.
Gallo, N., Natali, M. L., Quarta, A., Gaballo, A., Terzi, A., Sibillano, T., Giannini, C., De Benedetto, G. E., Lunetti, P., Capobianco, L., Blasi, F. S., Sicuro, A., Corallo, A., Sannino, A., & Salvatore, L. (2022). Aquaponics-Derived Tilapia Skin Collagen for Biomaterials Development. Polymers, 14(9). https://doi.org/10.3390/polym14091865
Gaspar-Pintiliescu, A., Anton, E. D., Iosageanu, A., Berger, D., Matei, C., Mitran, R., Negreanu-Pirjol, T., Craciunescu, O., & Moldovan, L. (2021). Enhanced Wound Healing Activity of Undenatured Type I Collagen Isolated from Discarded Skin of Black Sea Gilthead Bream (Sparus aurata) Conditioned as 3D Porous Dressing. Chemistry & Biodiversity, 18(8). https://doi.org/10.1002/cbdv.202100293
George, J., & Manjusha, W. (2020). Extraction and Purification of Collagen from Marine Squid Uroteuthis Duvauceli. International Journal of Life Science and Pharma Research, 10(4), 77–89. https://doi.org/10.22376/ijpbs/lpr.2020.10.4.L77-89
Georgieva, V., Zvezdova, D., & Vlaev, L. (2012). Non-isothermal kinetics of thermal degradation of chitosan. Chemistry Central Journal, 6(1), 81. https://doi.org/10.1186/1752-153X-6-81
Gibert, O., & Kumar Rakshit, S. (2005). Cassava starch snack formulation using functional shell fish by-products: mechanical, sorption and geometric properties. Journal of the Science of Food and Agriculture, 85(11), 1938–1946. https://doi.org/10.1002/jsfa.2200
Gomes, L. C., Faria, S. I., Valcarcel, J., Vázquez, J. A., Cerqueira, M. A., Pastrana, L., Bourbon, A. I., & Mergulhão, F. J. (2021). The effect of molecular weight on the antimicrobial activity of chitosan from Loligo opalescens for food packaging applications. Marine Drugs, 19(7). https://doi.org/10.3390/md19070384
Guerra, I. C. D., De Oliveira, P. D. L., Santos, M. M. F., Lúcio, A. S. S. C., Tavares, J. F., Barbosa-Filho, J. M., Madruga, M. S., & De Souza, E. L. (2016). The effects of composite coatings containing chitosan and Mentha

(piperita L. or x villosa Huds) essential oil on postharvest mold occurrence and quality of table grape cv. Isabella. Innovative Food Science and Emerging Technologies, 34, 112–121. https://doi.org/10.1016/j.ifset.2016.01.008
Guo, H., Hong, Z., & Yi, R. (2015). Core-Shell Collagen Peptide Chelated Calcium/Calcium Alginate Nanoparticles from Fish Scales for Calcium Supplementation. Journal of Food Science, 80(7), N1595–N1601. https://doi.org/10.1111/1750-3841.12912
Hajiali, F., Vidal, J., Jin, T. Y., de la Garza, L. C., Santos, M., Yang, G. L., & Moores, A. (2022). Extraction of Chitin from Green Crab Shells by Mechanochemistry and Aging. ACS SUSTAINABLE CHEMISTRY & ENGINEERING, 10(34), 11348–11357. https://doi.org/10.1021/acssuschemeng.2c02966
Halal, C. Y., Moura, J. M., & Pinto, L. A. A. (2011). Evaluation of molecular weight of chitosan in thin-layer and spouted bed drying. Journal of Food Process Engineering, 34(1), 160–174. https://doi.org/10.1111/j.1745-4530.2008.00345.x
Hammami, A., Hamdi, M., Abdelhedi, O., Jridi, M., Nasri, M., & Bayoudh, A. (2017). Surfactant- and oxidant-stable alkaline proteases from Bacillus invictae : Characterization and potential applications in chitin extraction and as a detergent additive. International Journal of Biological Macromolecules, 96, 272–281. https://doi.org/10.1016/j.ijbiomac.2016.12.035
Han, S. B., Won, B., Yang, S. chan, & Kim, D. H. (2021). Asterias pectinifera derived collagen peptide-encapsulating elastic nanoliposomes for the cosmetic application. Journal of Industrial and Engineering Chemistry, 98, 289–297. https://doi.org/10.1016/J.JIEC.2021.03.039
Hazeena, S. H., Hou, C.-Y., Zeng, J.-H., Li, B.-H., Lin, T.-C., Liu, C.-S., Chang, C.-I., Hsieh, S.-L., & Shih, M.-K. (2022). Extraction Optimization and Structural Characteristics of Chitosan from Cuttlefish (S. pharaonis sp.) Bone. Materials, 15(22). https://doi.org/10.3390/ma15227969
Hemalatha, T., UmaMaheswari, T., Senthil, R., Krithiga, G., & Anbukkarasi, K. (2017). Efficacy of chitosan films with basil essential oil: perspectives in food packaging. Journal of Food Measurement and Characterization, 11(4), 2160–2170. https://doi.org/10.1007/s11694-017-9601-7
Heng, T. T., Tey, J. Y., Soon, K. S., & Woo, K. K. (2022). Utilizing Fish Skin of Ikan Belida (Notopterus lopis) as a Source of Collagen: Production and Rheology Properties. Marine Drugs, 20(8). https://doi.org/10.3390/md20080525
Hofman, K., Tucker, N., Stanger, J., Staiger, M., Marshall, S., & Hall, B. (2012). Effects of the molecular format of collagen on characteristics of electrospun fibres. Journal of Materials Science, 47(3), 1148–1155. https://doi.org/10.1007/s10853-011-5775-2
Hou, E.-J., Hsieh, Y.-Y., Hsu, T.-W., Huang, C.-S., Lee, Y.-C., Han, Y.-S., & Chu, H.-T. (2022). Using the concept of circular economy to reduce the environmental impact of COVID-19 face mask waste. Sustainable Materials and Technologies, 33. https://doi.org/10.1016/j.susmat.2022.e00475

Huang, C.-Y., Kuo, C.-H., Wu, C.-H., Ku, M.-W., & Chen, P.-W. (2018). Extraction of crude chitosans from squid (Illex argentinus) pen by a compressional puffing-pretreatment process and evaluation of their antibacterial activity. Food Chemistry, 254, 217–223. https://doi.org/10.1016/j.foodchem.2018.02.018
Ilankovan, P., Hein, S., Ng, C.-H., Trung, T. S., & Stevens, W. F. (2006). Production of N-acetyl chitobiose from various chitin substrates using commercial enzymes. Carbohydrate Polymers, 63(2), 245–250. https://doi.org/10.1016/j.carbpol.2005.08.060
Jabeur, F., Mechri, S., Mensi, F., Gharbi, I., Naser, Y. B., Kriaa, M., Bejaoui, N., Bachouche, S., Badis, A., Annane, R., Djellali, M., Sadok, S., & Jaouadi, B. (2022). Extraction and characterization of chitin, chitosan, and protein hydrolysate from the invasive Pacific blue crab, Portunus segnis (Forskål, 1775) having potential biological activities. Environmental Science and Pollution Research, 29(24), 36023–36039. https://doi.org/10.1007/s11356-021-18398-y
Jadhav, U., & Pillai, A. (2011). Preparation and comparative study of chitosan from shells of different marine and freshwater prawns. Biosciences Biotechnology Research Asia, 8(2), 869–872. https://doi.org/10.13005/bbra/954
Jaiswal, M., Chauhan, D., & Sankararamakrishnan, N. (2012). Copper chitosan nanocomposite: Synthesis, characterization, and application in removal of organophosphorous pesticide from agricultural runoff. Environmental Science and Pollution Research, 19(6), 2055–2062. https://doi.org/10.1007/s11356-011-0699-6
Jeong, H.-S., Venkatesan, J., & Kim, S.-K. (2013). Isolation and characterization of collagen from marine fish (Thunnus obesus). Biotechnology and Bioprocess Engineering, 18(6), 1185–1191. https://doi.org/10.1007/s12257-013-0316-2
Jin, T., Liu, T., Jiang, S., Kurdyla, D., Klein, B. A., Michaelis, V. K., Lam, E., Li, J., & Moores, A. (2021). Chitosan nanocrystals synthesis: Via aging and application towards alginate hydrogels for sustainable drug release. Green Chemistry, 23(17), 6527–6537. https://doi.org/10.1039/d1gc01611c
Kim, S.-C., Heo, S.-Y., Oh, G.-W., Yi, M., & Jung, W.-K. (2022). A 3D-Printed Polycaprolactone/Marine Collagen Scaffold Reinforced with Carbonated Hydroxyapatite from Fish Bones for Bone Regeneration. Marine Drugs, 20(6). https://doi.org/10.3390/md20060344
Kimura, S. (1971). Studies On Marine Invertebrate Collagens—Iv: Structural Difference In Collagens From Cuticle And Intestines of Neanthes Diversicolor. NIPPON SUISAN GAKKAISHI, 37(5), 432–440. https://doi.org/10.2331/suisan.37.432
Kimura, S. (1971). Studies On Marine Invertebrate Collagens—III: Characterization of Cuticle Collagens In Annelids. NIPPON SUISAN GAKKAISHI, 37(5), 419–431. https://doi.org/10.2331/suisan.37.419
Kimura, S. (1969). Studies on Marine Invertebrate Collagens--II. Molecular Properties of Soluble Cuticle Collagen from a Marine Worm, Nereis japonica. NIPPON SUISAN GAKKAISHI, 35(11), 1121–1130. https://doi.org/10.2331/suisan.35.1121

Kimura, S., Nagaoka, Y., & Kubota, M. (1969). Studies on Marine Invertebrate Collagens—I. Some Collagens from Crustaceans and Molluscs. NIPPON SUISAN GAKKAISHI, 35(8), 743–748. https://doi.org/10.2331/suisan.35.743
Kubota, M., Kimura, S., & Kobayashi, N. (1972). Studies on Marine Invertebrate Collagens—VI. Some Properties of Collagen from an Ascidian Halocynthia roretzi. NIPPON SUISAN GAKKAISHI, 38(10), 1163–1169. https://doi.org/10.2331/suisan.38.1163
Kumaran, S., Perianaika Anahas, A. M., Prasannabalaji, N., Karthiga, M., Bharathi, S., Rajasekar, T., Joseph, J., Prasad, S. G., Pandian, S., Pugazhvendan, S. R., Pugazhvendan, S. R., & Aruni, W. (2021). Chitin derivatives of NAG and chitosan nanoparticles from marine disposal yards and their use for economically feasible fish feed development. Chemosphere, 281. https://doi.org/10.1016/j.chemosphere.2021.130746
Kumari, S., Rath, P., Sri Hari Kumar, A., & Tiwari, T. N. (2015). Extraction and characterization of chitin and chitosan from fishery waste by chemical method. Environmental Technology and Innovation, 3, 77–85. https://doi.org/10.1016/j.eti.2015.01.002
Laaraibi, A., Charhouf, I., Bennamara, A., Abourriche, A., & Berrada, M. (2015). Valorization of marine wastes in a preserving film based on chitosan for food applications. Journal of Materials and Environmental Science, 6(12), 3511–3516.
Lassoued, I., Hajji, S., Mhamdi, S., Jridi, M., Bayoudh, A., Barkia, A., & Nasri, M. (2015). Digestive alkaline proteases from thornback ray (Raja clavata): Characteristics and applications. International Journal of Biological Macromolecules, 80, 668–675. https://doi.org/10.1016/j.ijbiomac.2015.07.038
Lassoued, I., Mora, L., Barkia, A., Aristoy, M. C., Nasri, M., & Toldrá, F. (2015). Bioactive peptides identified in thornback ray skin's gelatin hydrolysates by proteases from Bacillus subtilis and Bacillus amyloliquefaciens. Journal of Proteomics, 128, 8–17. https://doi.org/10.1016/J.JPROT.2015.06.016
LeCorre-Bordes, D. S., Jaksons, P., & Hofman, K. (2017). Mind the gap: Ensuring laboratory-scale testing of an electrospinning product meets commercial-scale needs. Journal of Applied Polymer Science, 134(20). https://doi.org/10.1002/app.44836
Lee, J. E., Noh, S. K., & Kim, M. J. (2022). Effects of Enzymatic- and Ultrasound-Assisted Extraction on Physicochemical and Antioxidant Properties of Collagen Hydrolysate Fractions from Alaska Pollack (Theragra chalcogramma) Skin. ANTIOXIDANTS, 11(11). https://doi.org/10.3390/antiox11112112 WE - Science Citation Index Expanded (SCI-EXPANDED)
Liang, T.-W., Hsieh, J.-L., & Wang, S.-L. (2012). Production and purification of a protease, a chitosanase, and chitin oligosaccharides by Bacillus cereus TKU022 fermentation. Carbohydrate Research, 362, 38–46. https://doi.org/10.1016/j.carres.2012.08.004
Lima, M., Gomes, L. C., Teixeira-Santos, R., Romeu, M. J., Valcarcel, J., Vazquez, J. A., Cerqueira, M. A., Pastrana, L., Bourbon, A. I., de Jong, E. D., Sjollema, J., & Mergulhao, F. J. (2022). Assessment of the Antibiofilm Performance of Chitosan-Based Surfaces in Marine Environments. INTERNATIONAL JOURNAL OF MOLECULAR

SCIENCES, 23(23). https://doi.org/10.3390/ijms232314647 WE - Science Citation Index Expanded (SCI-EXPANDED)

Lin, F., Rong, H., Lin, J., Yuan, Y., Yu, J., Yu, C., You, C., Wang, S., Sun, Z., & Wen, X. (2020). Enhancement of collagen deposition in swim bladder of Chu's croaker (Nibea coibor) by proline: View from in-vitro and in-vivo study. Aquaculture, 523. https://doi.org/10.1016/j.aquaculture.2020.735175

Liu, D., Huang, J., Wu, C., Liu, C., Huang, R., Wang, W., Yin, T., Yan, X., He, H., & Chen, L. (2019). Purification, characterization, and application for preparation of antioxidant peptides of extracellular protease from Pseudoalteromonas sp. H2. Molecules, 24(18). https://doi.org/10.3390/molecules24183373

Lopes, C., Antelo, L. T., Franco-Uría, A., Alonso, A. A., & Pérez-Martín, R. (2018). Chitin production from crustacean biomass: Sustainability assessment of chemical and enzymatic processes. Journal of Cleaner Production, 172, 4140–4151. https://doi.org/10.1016/j.jclepro.2017.01.082

Machałowski, T., Czajka, M., Petrenko, I., Meissner, H., Schimpf, C., Rafaja, D., Ziętek, J., Dzięgiel, B., Adaszek, Ł., Voronkina, A., Kovalchuk, V., Jaroszewicz, J., Fursov, A., Rahimi-Nasrabadi, M., Stawski, D., Bechmann, N., Jesionowski, T., & Ehrlich, H. (2020). Functionalization of 3D Chitinous Skeletal Scaffolds of Sponge Origin Using Silver Nanoparticles and Their Antibacterial Properties. Marine Drugs, 18(6), 304. https://doi.org/10.3390/md18060304

Maeda, H., Hosomi, R., Chiba, U., & Fukunaga, K. (2013). Chemical composition of salmon ovary outer membrane and its protein increases fecal mucins content in c57bl/6j and type 2 diabetic/obese kk-ay mice. Foods, 2(3), 415–429. https://doi.org/10.3390/foods2030415

Magnacca, G., Guerretta, F., Vizintin, A., Benzi, P., Valsania, M. C., & Nisticò, R. (2018). Preparation, characterization and environmental/electrochemical energy storage testing of low-cost biochar from natural chitin obtained via pyrolysis at mild conditions. Applied Surface Science, 427, 883–893. https://doi.org/10.1016/j.apsusc.2017.07.277

Manni, L., Ghorbel-Bellaaj, O., Jellouli, K., Younes, I., & Nasri, M. (2010). Extraction and characterization of chitin, chitosan, and protein hydrolysates prepared from shrimp waste by treatment with crude protease from bacillus cereus SV1. Applied Biochemistry and Biotechnology, 162(2), 345–357. https://doi.org/10.1007/s12010-009-8846-y

Marimuthu, R., Devarayan, K., Sukumaran, M., Suresh, A., & Ravichandran, S. (2022). Piezoelectric property from processed crustacean shells. Materials Today: Proceedings, 58, 942–946. https://doi.org/10.1016/j.matpr.2021.12.402

Martins, E., Diogo, G. S., Pires, R., Reis, R. L., & Silva, T. H. (2022). 3D Biocomposites Comprising Marine Collagen and Silica-Based Materials Inspired on the Composition of Marine Sponge Skeletons Envisaging Bone Tissue Regeneration. Marine Drugs, 20(11). https://doi.org/10.3390/md20110718

Martins, E., Fernandes, R., Alves, A. L., Sousa, R. O., Reis, R. L., & Silva, T. H. (2022). Skin Byproducts of Reinhardtius hippoglossoides (Greenland Halibut) as Ecosustainable Source of Marine Collagen. Applied Sciences (Switzerland), 12(21). https://doi.org/10.3390/app122111282

Mathew, G. M., Puthiyamadam, A., Sasikumar, K., Ashoor, S., & Sukumaran, R. K. (2021). Biological treatment of prawn shell wastes for valorization and waste management. Bioresource Technology Reports, 15. https://doi.org/10.1016/j.biteb.2021.100788
Maulani, D., Nofianti, K., Sugijanto, N., & Kartosentono, S. (2021). An Eco-Friendly Absorption Method of Cu2+, Cd2+, and Pb2+ Using the Shells and Chitosan Derived from Solen vagina. Journal of Ecological Engineering, 22(7), 212–222. https://doi.org/10.12911/22998993/139118
Mauro, M., Pinto, P., Settanni, L., Puccio, V., Vazzana, M., Hornsby, B. L., Fabbrizio, A., Di Stefano, V., Barone, G., & Arizza, V. (2022). Chitosan Film Functionalized with Grape Seed Oil-Preliminary Evaluation of Antimicrobial Activity. SUSTAINABILITY, 14(9). https://doi.org/10.3390/su14095410 WE - Science Citation Index Expanded (SCI-EXPANDED) WE - Social Science Citation Index (SSCI)
McReynolds, C., Adrien, A., de Fraissinette, N. B., Olza, S., & Fernandes, S. C. M. (2022). Deep eutectic solvents for the extraction of β-chitin from Loligo vulgaris squid pens: a sustainable way to valorize fishery by-products. Biomass Conversion and Biorefinery. https://doi.org/10.1007/s13399-022-03569-9
Melotti, L., Martinello, T., Perazzi, A., Iacopetti, I., Ferrario, C., Sugni, M., Sacchetto, R., & Patruno, M. (2021). A Prototype Skin Substitute, Made of Recycled Marine Collagen, Improves the Skin Regeneration of Sheep. Animals, 11(5), 1219. https://doi.org/10.3390/ani11051219
Merz, C. R. (2019). Physicochemical and Colligative Investigation of α (Shrimp Shell)- And β (Squid Pen)-Chitosan Membranes: Concentration-Gradient-Driven Water Flux and Ion Transport for Salinity Gradient Power and Separation Process Operations. ACS Omega, 4(25), 21027–21040. https://doi.org/10.1021/acsomega.9b02357
Miron, A., Sarbu, A., Zaharia, A., Sandu, T., Iovu, H., Fierascu, R. C., Neagu, A. L., Chiriac, A. L., & Iordache, T. V. (2022). A Top-Down Procedure for Synthesizing Calcium Carbonate-Enriched Chitosan from Shrimp Shell Wastes. GELS, 8(11). https://doi.org/10.3390/gels8110742 WE - Science Citation Index Expanded (SCI-EXPANDED)
Mittal, A., Singh, A., Aluko, R. E., & Benjakul, S. (2021). Pacific white shrimp (Litopenaeus vannamei) shell chitosan and the conjugate with epigallocatechin gallate: Antioxidative and antimicrobial activities. Journal of Food Biochemistry, 45(1). https://doi.org/10.1111/jfbc.13569
Mizuta, S., Yoshinaka, R., Sato, M., & Sakaguchi, M. (1994). Characterization of Collagen in the Muscle of Several Crustacean Species in Association with Raw Meat Texture. Fisheries Science, 60(3), 323–328. https://doi.org/10.2331/fishsci.60.323
Mohanasrinivasan, V., Mishra, M., Paliwal, J. S., Singh, S. K., Selvarajan, E., Suganthi, V., & Subathra Devi, C. (2014). Studies on heavy metal removal efficiency and antibacterial activity of chitosan prepared from shrimp shell waste. 3 Biotech, 4(2), 167–175. https://doi.org/10.1007/s13205-013-0140-6
Moreno-Sader, K. A., Martinez-Consuegra, J. D., & González-Delgado, Á. D. (2021). Development of a biorefinery approach for shrimp processing in North-Colombia: Process simulation and sustainability assessment. Environmental Technology & Innovation, 22, 101461. https://doi.org/10.1016/j.eti.2021.101461

Morganti, P. (2016). New horizon in cosmetic dermatology. Journal of Applied Cosmetology, 34(1–2), 15–24.
Morganti, P. (2015). Innovative and sustainable bio-polymers for household and beauty care products. Final results of the EU BIO-MIMETIC Project. Journal of Applied Cosmetology, 33(1–2), 67–84.
Mushi, N. E., Kochumalayil, J., Cervin, N. T., Zhou, Q., & Berglund, L. A. (2016). Nanostructurally Controlled Hydrogel Based on Small-Diameter Native Chitin Nanofibers: Preparation, Structure, and Properties. ChemSusChem, 9(9), 989–995. https://doi.org/10.1002/cssc.201501697
Muthukumar, T., Prakash, D., Anbarasu, K., Kumar, B. S., & Sastry, T. P. (2014). Effect of collagen sponge incorporating Macrotyloma uniflorum extract on full-thickness wound healing by down-regulation of matrix metalloproteinases and inflammatory markers. RSC Advances, 4(109), 64267–64276. https://doi.org/10.1039/c4ra11959b
Muthumari, K., Anand, M., & Maruthupandy, M. (2016). Collagen Extract from Marine Finfish Scales as a Potential Mosquito Larvicide. Protein Journal, 35(6), 391–400. https://doi.org/10.1007/s10930-016-9685-7
Nerdy, N., Lestari, P., Simorangkir, D., Aulianshah, V., Yusuf, F., & Bakri, T. K. (2022). COMPARISON OF CHITOSAN FROM CRAB SHELL WASTE AND SHRIMP SHELL WASTE AS NATURAL ADSORBENT AGAINST HEAVY METALS AND DYES. International Journal of Applied Pharmaceutics, 14(2), 181–185. https://doi.org/10.22159/ijap.2022v14i2.43560
Nessa, F., Khan, S. A., & Al Khatib, F. M. A. (2010). Production and characterization of chitosan from shrimp (Penaeus semisulcatus) shell waste of UAE. Pakistan Journal of Scientific and Industrial Research, 53(1), 52–58.
Nishimoto, M., Mizuta, S., Yoshinaka, R., Park, E. Y., Nakamura, Y., & Sato, K. (2009). Characterization and Comparison of Collagens Extracted from the Digestive Tract and Skin of a Japanese Amberjack Seriola Quinqueradiata. Journal of Food Biochemistry, 33(6), 777–789. https://doi.org/10.1111/j.1745-4514.2009.00250.x
Nunes, C., Maricato, É., Cunha, Â., Rocha, M. A. M., Santos, S., Ferreira, P., Silva, M. A., Rodrigues, A., Amado, O., Coimbra, J., Silva, D., Moreira, A., Mendo, S., Lopes da Silva, J. A., Pereira, E., Rocha, S. M., & Coimbra, M. A. (2016). Chitosan–genipin film, a sustainable methodology for wine preservation. Green Chemistry, 18(19), 5331–5341. https://doi.org/10.1039/C6GC01621A
Omar, B. A., Elmasry, R., Eita, A., Soliman, M. M., El-Tahan, A. M., & Sitohy, M. (2022). Upgrading the preparation of high-quality chitosan from Procambarus clarkii wastes over the traditional isolation of shrimp chitosan. SAUDI JOURNAL OF BIOLOGICAL SCIENCES, 29(2), 911–919. https://doi.org/10.1016/j.sjbs.2021.10.014 WE - Science Citation Index Expanded (SCI-EXPANDED)
Omidinasab, M., Rahbar, N., Ahmadi, M., Kakavandi, B., Ghanbari, F., Kyzas, G. Z., Martinez, S. S., & Jaafarzadeh, N. (2018). Removal of vanadium and palladium ions by adsorption onto magnetic chitosan nanoparticles. Environmental Science and Pollution Research, 25(34), 34262–34276. https://doi.org/10.1007/s11356-018-3137-1

Pachapur, V. L., Guemiza, K., Rouissi, T., Sarma, S. J., & Brar, S. K. (2016). Novel biological and chemical methods of chitin extraction from crustacean waste using saline water. Journal of Chemical Technology and Biotechnology, 91(8), 2331–2339. https://doi.org/10.1002/jctb.4821

Pacheco, N., Garnica-Gonzalez, M., Gimeno, M., Bárzana, E., Trombotto, S., David, L., & Shirai, K. (2011). Structural characterization of chitin and chitosan obtained by biological and chemical methods. Biomacromolecules, 12(9), 3285–3290. https://doi.org/10.1021/bm200750t

Paduretu, C.-C., Apetroaei, M. R., Apetroaei, G. M., Atodiresei, D. V., & Rau, I. (2020). Dyes adsorption by using different types of chitosan for decontamination of cleaning waters from chemical carriers. Journal of Environmental Protection and Ecology, 21(1), 28–36.

Pădurețu, C.-C., Apetroaei, M. R., Rău, I., & Schroder, V. (2018). Characterization of chitosan extracted from different romanian black sea crustaceans. UPB Scientific Bulletin, Series B: Chemistry and Materials Science, 80(3), 13–24.

Pallela, R., Bojja, S., & Janapala, V. R. (2011). Biochemical and biophysical characterization of collagens of marine sponge, Ircinia fusca (Porifera: Demospongiae: Irciniidae). International Journal of Biological Macromolecules, 49(1), 85–92. https://doi.org/10.1016/j.ijbiomac.2011.03.019

Palma, G., Casals, P., & Cardenas, G. (2005). Synthesis and characterization of new chitosan-O-ethyl phosphonate. Journal of the Chilean Chemical Society, 50(4), 719–724. https://doi.org/10.4067/s0717-97072005000400013

Pandara, D. P., Masengi, K. W. A., Tamuntuan, G. H., Angmalisang, P. A., Wuntu, A. D., Ferdy, F., Bobanto, M. D., & Sompotan, A. F. (2022). The potential of fish scale application as photothermal raw material in seawater desalination. AACL Bioflux, 15(4), 1617–1629.

Paradiso, F., Fitzgerald, J., Yao, S., Barry, F., Taraballi, F., Gonzalez, D., Conlan, R. S., & Francis, L. (2019). Marine Collagen Substrates for 2D and 3D Ovarian Cancer Cell Systems. Frontiers in Bioengineering and Biotechnology, 7. https://doi.org/10.3389/fbioe.2019.00343

Pati, S., Sarkar, T., Sheikh, H. I., Bharadwaj, K. K., Mohapatra, P. K., Chatterji, A., Dash, B. P., Edinur, H. A., & Nelson, B. R. (2021). γ-Irradiated Chitosan From Carcinoscorpius rotundicauda (Latreille, 1802) Improves the Shelf Life of Refrigerated Aquatic Products. Frontiers in Marine Science, 8. https://doi.org/10.3389/fmars.2021.664961

Paul, T., Halder, S. K., Das, A., Ghosh, K., Mandal, A., Payra, P., Barman, P., Das Mohapatra, P. K., Pati, B. R., & Mondal, K. C. (2015). Production of chitin and bioactive materials from Black tiger shrimp (Penaeus monodon) shell waste by the treatment of bacterial protease cocktail. 3 Biotech, 5(4), 483–493. https://doi.org/10.1007/s13205-014-0245-6

Phuong, P. T. D., Trung, T. S., Stevens, W. F., Minh, N. C., Bao, H. N. D., & Hoa, N. V. (2021). Valorization of Heavy Waste of Modern Intensive Shrimp Farming as a Potential Source for Chitin and Chitosan Production. Waste and Biomass Valorization, 13(2), 823–830. https://doi.org/10.1007/s12649-021-01557-0

Prelipcean, A.-M., Iosageanu, A., Gaspar-Pintiliescu, A., Moldovan, L., Craciunescu, O., Negreanu-Pirjol, T., Negreanu-Pirjol, B., Mitran, R.-A., Marin, M., & D'Amora, U. (2022). Marine and Agro-Industrial By-Products Valorization Intended for Topical Formulations in Wound Healing Applications. Materials, 15(10). https://doi.org/10.3390/ma15103507
Rahali, A., Riazi, A., Moussaoui, B., Boucherdoud, A., & Bektaş, N. (2020). Decolourisation of methylene blue and congo red dye solutions by adsorption using chitosan. Desalination and Water Treatment, 198, 422–433. https://doi.org/10.5004/dwt.2020.26093
Rashid, T. U., Rahman, M. M., Kabir, S., Shamsuddin, S. M., & Khan, M. A. (2012). A new approach for the preparation of chitosan from γ-irradiation of prawn shell: Effects of radiation on the characteristics of chitosan. Polymer International, 61(8), 1302–1308. https://doi.org/10.1002/pi.4207
Rastian, Z., Pütz, S., Wang, Y., Kumar, S., Fleissner, F., Weidner, T., & Parekh, S. H. (2018). Type I Collagen from Jellyfish Catostylus mosaicus for Biomaterial Applications. ACS Biomaterials Science and Engineering, 4(6), 2115–2125. https://doi.org/10.1021/acsbiomaterials.7b00979
Rethinam, S., Nivedita, P., Hemalatha, T., Vedakumari, S. W., & Sastry, T. P. (2016). A possible wound dressing material from marine food waste. International Journal of Artificial Organs, 39(10), 509–517. https://doi.org/10.5301/ijao.5000531
Rissouli, L., Benicha, M., & Chabbi, M. (2016). Contribution to the elimination of Linuron by the adsorption process using Chitin and Chitosan biopolymers. Journal of Materials and Environmental Science, 7(2), 531–540.
Rizzi, V., Gubitosa, J., Fini, P., Romita, R., Nuzzo, S., & Cosma, P. (2019). Chitosan biopolymer from crab shell as recyclable film to remove/recover in batch ketoprofen from water: Understanding the factors affecting the adsorption process. Materials, 12(23). https://doi.org/10.3390/ma122333810
Rodríguez, F., Morán, L., González, G., Troncoso, E., & Zúñiga, R. N. (2017). Collagen extraction from mussel byssus: a new marine collagen source with physicochemical properties of industrial interest. Journal of Food Science and Technology, 54(5), 1228–1238. https://doi.org/10.1007/s13197-017-2566-z
Rodriguez-Veiga, I., Acosta, N., Aranaz, I., & Dobrzycka-Krahel, A. (2022). Exploring Saduria entomon (Crustacea Isopoda) as a New Source for Chitin and Chitosan Isolation. International Journal of Molecular Sciences, 23(24). https://doi.org/10.3390/ijms232416125
Rong, H., Lin, F., Ning, L., Wu, K., Chen, B., Zheng, J., Limbu, S. M., & Wen, X. (2022). Cloning, tissue distribution and mRNA expression of type I collagen alpha 1 gene from Chu's croaker (Nibea coibor). Gene, 824, 146441. https://doi.org/10.1016/j.gene.2022.146441
Rumengan, I. F. M., Suptijah, P., Wullur, S., & Talumepa, A. (2017). Characterization of chitin extracted from fish scales of marine fish species purchased from local markets in North Sulawesi, Indonesia. IOP Conference Series: Earth and Environmental Science, 89, 012028. https://doi.org/10.1088/1755-1315/89/1/012028

Salazar, R., Salas-Gomez, V., Alvarado, A. A., & Baykara, H. (2022). Preparation, Characterization and Evaluation of Antibacterial Properties of Polylactide-Polyethylene Glycol-Chitosan Active Composite Films. Polymers, 14(11). https://doi.org/10.3390/polym14112266
Salazar-Leyva, J. A., Lizardi-Mendoza, J., Ramirez-Suarez, J. C., Valenzuela-Soto, E. M., Ezquerra-Brauer, J. M., Castillo-Yañez, F. J., & Pacheco-Aguilar, R. (2013). Acidic proteases from monterey sardine (Sardinops sagax caerulea) Immobilized on shrimp waste chitin and chitosan supports: Searching for a by-product catalytic system. Applied Biochemistry and Biotechnology, 171(3), 795–805. https://doi.org/10.1007/s12010-013-0407-8
Samiei, M. H., Jamili, S., Nikukar, H., & Razban, V. (2022). Isolation, characterization and biocompatibility evaluation of collagen from Thunnus tonggol skin. IRANIAN JOURNAL OF FISHERIES SCIENCES, 21(2), 568–589. https://doi.org/10.22092/ijfs.2022.126579 WE - Science Citation Index Expanded (SCI-EXPANDED)
Sánchez-Duarte, R. G., Sánchez-Machado, D. I., López-Cervantes, J., & Correa-Murrieta, M. A. (2012). Adsorption of allura red dye by cross-linked chitosan from shrimp waste. Water Science and Technology, 65(4), 618–623. https://doi.org/10.2166/wst.2012.900
Sanz, B., Sanchez, A. A., Tangey, B., Gilmore, K., Yue, Z., Liu, X., & Wallace, G. (2021). Light cross-linkable marine collagen for coaxial printing of a 3D model of neuromuscular junction formation. Biomedicines, 9(1), 1–19. https://doi.org/10.3390/biomedicines9010016
Sari, E., Herawati, Anshori, U., & Nurmayulis. (2019). Biocoagulant of blood based on chitosan nanoparticle from crustacea. Journal of Physics: Conference Series, 1246(1). https://doi.org/10.1088/1742-6596/1246/1/012055
Seixas, M. J., Martins, E., Reis, R. L., & Silva, T. H. (2020). Extraction and Characterization of Collagen from Elasmobranch Byproducts for Potential Biomaterial Use. Marine Drugs, 18(12). https://doi.org/10.3390/md18120617
Selvakumar, G., Kuttalam, I., Mukundan, S., & Lonchin, S. (2021). Valorization of toxic discarded fish skin for biomedical application. Journal of Cleaner Production, 323. https://doi.org/10.1016/j.jclepro.2021.129147
Shamshina, J. L., & Abidi, N. (2022). Isolation of Chitin Nano-whiskers Directly from Crustacean Biomass Waste in a Single Step with Acidic Ionic Liquids. ACS SUSTAINABLE CHEMISTRY & ENGINEERING, 10(36), 11846–11855. https://doi.org/10.1021/acssuschemeng.2c02461
Siddiqui, Y. D., Arief, E. M., Yusoff, A., Suzina, A. H., & Abdullah, S. Y. (2013). Isolation of pepsin-solubilized collagen (PSC) from crude collagen extracted from body wall of sea cucumber (Bohadschia spp.). International Journal of Pharmacy and Pharmaceutical Sciences, 5(SUPPL. 2), 555–559.
Sila, A., Mlaik, N., Sayari, N., Balti, R., & Bougatef, A. (2014). Chitin and Chitosan Extracted from Shrimp Waste Using Fish Proteases Aided Process: Efficiency of Chitosan in the Treatment of Unhairing Effluents. Journal of Polymers and the Environment, 22(1), 78–87. https://doi.org/10.1007/s10924-013-0598-7
Silva, J. C., Barros, A. A., Aroso, I. M., Fassini, D., Silva, T. H., Reis, R. L., & Duarte, A. R. C. (2016). Extraction of Collagen/Gelatin from the Marine Demosponge Chondrosia reniformis (Nardo, 1847) Using Water Acidified

with Carbon Dioxide - Process Optimization. Industrial and Engineering Chemistry Research, 55(25), 6922–6930. https://doi.org/10.1021/acs.iecr.6b00523

Silva, R. S. G., Bandeira, S. F., & Pinto, L. A. A. (2014). Characteristics and chemical composition of skins gelatin from cobia (Rachycentron canadum). LWT, 57(2), 580–585. https://doi.org/10.1016/j.lwt.2014.02.026

Singh, A., Benjakul, S., & Prodpran, T. (2019). Ultrasound-Assisted Extraction of Chitosan from Squid Pen: Molecular Characterization and Fat Binding Capacity. Journal of Food Science, 84(2), 224–234. https://doi.org/10.1111/1750-3841.14439

Sinha, S., Tripathi, P., & Chand, S. (2012). A New Bifunctional Chitosanase Enzyme from Streptomyces sp. and Its Application in Production of Antioxidant Chitooligosaccharides. Applied Biochemistry and Biotechnology, 167(5), 1029–1039. https://doi.org/10.1007/s12010-012-9546-6

Sivakami, M. S., Gomathi, T., Venkatesan, J., Jeong, H.-S., Kim, S.-K., & Sudha, P. N. (2013). Preparation and characterization of nano chitosan for treatment wastewaters. International Journal of Biological Macromolecules, 57, 204–212. https://doi.org/10.1016/j.ijbiomac.2013.03.005

Sivakumar, P., Arichandran, R., Suguna, L., Mariappan, M., & Chandrakasan, G. (2000). The composition and characteristics of skin and muscle collagens from a freshwater catfish grown in biologically treated tannery effluent water. Journal of Fish Biology, 56(4), 999–1012. https://doi.org/10.1006/jfbi.1999.1225

Song, E., Yeon Kim, S., Chun, T., Byun, H.-J., & Lee, Y. M. (2006). Collagen scaffolds derived from a marine source and their biocompatibility. Biomaterials, 27(15), 2951–2961. https://doi.org/10.1016/j.biomaterials.2006.01.015

Song, X., Zhang, B., Cao, Y., Liu, B., & Chen, B. (2022). Shrimp-waste based dispersant as oil spill treating agent: Biodegradation of dispersant and dispersed oil. Journal of Hazardous Materials, 439, 129617. https://doi.org/10.1016/j.jhazmat.2022.129617

Sousa, R. O., Alves, A. L., Carvalho, D. N., Martins, E., Oliveira, C., Silva, T. H., & Reis, R. L. (2020). Acid and enzymatic extraction of collagen from Atlantic cod (Gadus Morhua) swim bladders envisaging health-related applications. Journal of Biomaterials Science, Polymer Edition, 31(1), 20–37. https://doi.org/10.1080/09205063.2019.1669313

Sun, T.-C., Yan, B.-Y., Ning, X.-C., Tang, Z.-Y., Hui, C., Hu, M.-Z., Ramakrishna, S., Long, Y.-Z., & Zhang, J. (2022). A nanofiber hydrogel derived entirely from ocean biomass for wound healing. Nanoscale Advances, 5(1), 160–170. https://doi.org/10.1039/d2na00535b

Sun, W. -Q., Payne, G. F., Moas, M. S. G. L., Chu, J. H., & Wallace, K. K. (1992). Tyrosinase Reaction/Chitosan Adsorption for Removing Phenols from Wastewater. Biotechnology Progress, 8(3), 179–186. https://doi.org/10.1021/bp00015a002

Tan, Y. N., Lee, P. P., & Chen, W. N. (2020). Microbial extraction of chitin from seafood waste using sugars derived from fruit waste-stream. AMB Express, 10(1). https://doi.org/10.1186/s13568-020-0954-7

Thomas, R., Fukamizo, T., & Suginta, W. (2022). Bioeconomic production of high-quality chitobiose from chitin food wastes using an in-house chitinase from Vibrio campbellii. Bioresources and Bioprocessing, 9(1), 86. https://doi.org/10.1186/s40643-022-00574-8

Tran, T. N., Doan, C. T., Nguyen, M. T., Nguyen, V. B., Vo, T. P. K., Nguyen, A. D., & Wang, S.-L. (2019). An Exochitinase with N-Acetyl-β-Glucosaminidase-Like Activity from Shrimp Head Conversion by Streptomyces speibonae and Its Application in Hydrolyzing β-Chitin Powder to Produce N-Acetyl-d-Glucosamine. Polymers, 11(10), 1600. https://doi.org/10.3390/polym11101600

Trung, T. S., Tram, L. H., Van Tan, N., Van Hoa, N., Minh, N. C., Loc, P. T., & Stevens, W. F. (2020). Improved method for production of chitin and chitosan from shrimp shells. Carbohydrate Research, 489, 107913. https://doi.org/10.1016/j.carres.2020.107913

Tzeng, T.-W., Bhaumik, P., & Chung, P.-W. (2019). Understanding the production of 5-hydroxymethylfurfural (HMF) from chitosan using solid acids. Molecular Catalysis, 479, 110627. https://doi.org/10.1016/j.mcat.2019.110627

Tziveleka, L.-A., Kikionis, S., Karkatzoulis, L., Bethanis, K., Roussis, V., & Ioannou, E. (2022). Valorization of Fish Waste: Isolation and Characterization of Acid- and Pepsin-Soluble Collagen from the Scales of Mediterranean Fish and Fabrication of Collagen-Based Nanofibrous Scaffolds. Marine Drugs, 20(11). https://doi.org/10.3390/md20110664

Uranga, J., Etxabide, A., Cabezudo, S., de la Caba, K., & Guerrero, P. (2020). Valorization of marine-derived biowaste to develop chitin/fish gelatin products as bioactive carriers and moisture scavengers. Science of The Total Environment, 706, 135747. https://doi.org/10.1016/j.scitotenv.2019.135747

Valcarcel, J., Fraguas, J., Hermida-Merino, C., Hermida-Merino, D., Piñeiro, M. M., & Vázquez, J. A. (2021). Production and physicochemical characterization of gelatin and collagen hydrolysates from turbot skin waste generated by aquaculture activities. Marine Drugs, 19(9). https://doi.org/10.3390/md19090491

Veeruraj, A., Arumugam, M., & Balasubramanian, T. (2013). Isolation and characterization of thermostable collagen from the marine eel-fish (Evenchelys macrura). Process Biochemistry, 48(10), 1592–1602. https://doi.org/10.1016/j.procbio.2013.07.011

Vendramin, V., Spinato, G., & Vincenzi, S. (2021). Shellfish chitosan potential in wine clarification. Applied Sciences (Switzerland), 11(10). https://doi.org/10.3390/app11104417

Venkatachalam, A., Govinda Rajulu, M. B., Thirunavukkarasu, N., & Suryanarayanan, T. S. (2015). Endophytic fungi of marine algae and seagrasses: A novel source of chitin modifying enzymes. Mycosphere, 6(3), 345–355. https://doi.org/10.5943/MYCOSPHERE/6/3/10

Vishnuprasad, & Senthil Kumar, K. (2015). Adsorption studies on treatment of cooking oil mill effluent using crab shell chitosan. Journal of Chemical and Pharmaceutical Research, 7(11), 19–29.

Wada, S., Ichikawa, H., & Tatsumi, K. (1993). Removal of phenols from wastewater by soluble and immobilized tyrosinase. Biotechnology and Bioengineering, 42(7), 854–858. https://doi.org/10.1002/bit.260420710
Wang, Y. H., Yang, Y. Q., Wang, R., Zhu, Y. L., Yang, P. B., Lin, Z. N., Wang, Z. H., & Cong, W. (2022). Effectively inhibiting the degradation of chitin during extraction from crustacean waste via a novel deep eutectic solvent aqueous solution. PROCESS BIOCHEMISTRY, 121, 142–151. https://doi.org/10.1016/j.procbio.2022.06.029
Wang, Y. H., Yang, Y. Q., Wang, R., Zhu, Y. L., Yang, P. B., Lin, Z. N., Wang, Z. H., & Cong, W. (2022). Efficient extraction of chitin from crustacean waste via a novel ternary natural deep eutectic solvents. CARBOHYDRATE POLYMERS, 286. https://doi.org/10.1016/j.carbpol.2022.119281
Wegner, L., Kinoshita, A., de Paiva, F. F. G., de Almeida Soares, P. N., Santana, W., & Pinto, E. M. (2021). Only carapace or the entire cephalothorax: which is best to obtain chitosan from shrimp fishery waste? Journal of Material Cycles and Waste Management, 23(5), 1831–1837. https://doi.org/10.1007/s10163-021-01254-z
Widyastuti, W., Setiawan, F., Al Afandy, C., Irawan, A., Laila, A., Juliasih, N. L. G. R., Setiawan, W. A., Arai, M., Hendri, J., & Setiawan, A. (2022). Antifungal Agent Chitooligosaccharides Derived from Solid-State Fermentation of Shrimp Shell Waste by Pseudonocardia antitumoralis 18D36-A1. Fermentation, 8(8), 353. https://doi.org/10.3390/fermentation8080353
Wisser, D., Wisser, F. M., Raschke, S., Klein, N., Leistner, M., Grothe, J., Brunner, E., & Kaskel, S. (2015). Biological Chitin-MOF Composites with Hierarchical Pore Systems for Air-Filtration Applications. Angewandte Chemie International Edition, 54(43), 12588–12591. https://doi.org/10.1002/anie.201504572
Wu, F.-C., Tseng, R.-L., & Juang, R.-S. (2000). Comparative adsorption of metal and dye on flake- and bead-types of chitosans prepared from fishery wastes. Journal of Hazardous Materials, 73(1), 63–75. https://doi.org/10.1016/S0304-3894(99)00168-5
Xu, Y., Bajaj, M., Schneider, R., Grage, S. L., Ulrich, A. S., Winter, J., & Gallert, C. (2013). Transformation of the matrix structure of shrimp shells during bacterial deproteination and demineralization. Microbial Cell Factories, 12(1). https://doi.org/10.1186/1475-2859-12-90
Xu, Y. J., Han, X. L., & Li, Y. (2010). Effect of marine collagen peptides on long bone development in growing rats. Journal of the Science of Food and Agriculture, 90(9), 1485–1491. https://doi.org/10.1002/jsfa.3972
Yamamoto, K., Yoshizawa, Y., Yanagiguchi, K., Ikeda, T., Yamada, S., & Hayashi, Y. (2015). The Characterization of Fish (Tilapia) Collagen Sponge as a Biomaterial. International Journal of Polymer Science, 2015. https://doi.org/10.1155/2015/957385
Yang, F., Jin, S., & Tang, Y. (2019). Marine collagen peptides promote cell proliferation of NIH-3T3 fibroblasts via NF-κB signaling pathway. Molecules, 24(22). https://doi.org/10.3390/molecules24224201
Youcefi, F., Ouahab, L. W., Borsali, L., & Bengherbi, S. E. (2022). Heavy metal removal efficiency and antibacterial activity of chitosan beads prepared from crustacean waste. In MATERIALS TODAY-PROCEEDINGS (Vol. 53,

Issue 11th Conference on Solid State Surfaces and Interfaces, pp. 265–268). https://doi.org/10.1016/j.matpr.2022.01.089

Zhang, H., Yu, H., Qian, Y., & Chen, S. (2017). Production of chitin & chitosan using successive three-step microbial fermentation. Journal of Polymer Materials, 34(1), 123–127.

Zhang, H., Yun, S., Song, L., Zhang, Y., & Zhao, Y. (2017). The preparation and characterization of chitin and chitosan under large-scale submerged fermentation level using shrimp by-products as substrate. International Journal of Biological Macromolecules, 96, 334–339. https://doi.org/10.1016/j.ijbiomac.2016.12.017

Zhang, J., Duan, R., Huang, L., Song, Y., & Regenstein, J. M. (2014). Characterisation of acid-soluble and pepsin-solubilised collagen from jellyfish (Cyanea nozakii Kishinouye). Food Chemistry, 150, 22–26. https://doi.org/10.1016/j.foodchem.2013.10.116

Zhang, Y., Zhou, Z., Liu, Y., Cao, Y., He, S., Huo, F., Qin, C., Yao, B., & Ringø, E. (2014). High-yield production of a chitinase from Aeromonas veronii B565 as a potential feed supplement for warm-water aquaculture. Applied Microbiology and Biotechnology, 98(4), 1651–1662. https://doi.org/10.1007/s00253-013-5023-6

Zhang, Z., Wang, J., Ding, Y., Dai, X., & Li, Y. (2011). Oral administration of marine collagen peptides from Chum Salmon skin enhances cutaneous wound healing and angiogenesis in rats. Journal of the Science of Food and Agriculture, 91(12), 2173–2179. https://doi.org/10.1002/jsfa.4435

Zhao, Y., & Li, J. (2016). Ascidian bioresources: Common and variant chemical compositions and exploitation strategy - Examples of Halocynthia roretzi, Styela plicata, Ascidia sp. and Ciona intestinalis. Zeitschrift Fur Naturforschung - Section C Journal of Biosciences, 71(5–6), 165–180. https://doi.org/10.1515/znc-2016-0012

Zheng, J., Tian, X., Xu, B., Yuan, F., Gong, J., & Yang, Z. (2020). Collagen peptides from swim bladders of giant croaker (Nibea japonica) and their protective effects against H2O2-induced oxidative damage toward human umbilical vein endothelial cells. Marine Drugs, 18(8). https://doi.org/10.3390/MD18080430

Zuorro, A., Moreno-Sader, K. A., & González-Delgado, Á. D. (2021). Evaluating the feasibility of a pilot-scale shrimp biorefinery via techno-economic analysis. Journal of Cleaner Production, 320. https://doi.org/10.1016/j.jclepro.2021.128740

Zuorro, A., Moreno-Sader, K. A. K. A. K. A., & González-Delgado, Á. D. Á. D. (2020). Economic Evaluation and Techno-Economic Sensitivity Analysis of a Mass Integrated Shrimp Biorefinery in North Colombia. Polymers, 12(10), 1–14. https://doi.org/10.3390/polym12102397
